# Circulating miRNAs Associated With ER Stress and Organ Damage in a Preclinical Model of Trauma Hemorrhagic Shock

**DOI:** 10.3389/fmed.2020.568096

**Published:** 2020-09-24

**Authors:** Andreia Luís, Matthias Hackl, Mohammad Jafarmadar, Claudia Keibl, Julia M. Jilge, Johannes Grillari, Soheyl Bahrami, Andrey V. Kozlov

**Affiliations:** ^1^Ludwig Boltzmann Institute for Experimental and Clinical Traumatology, Vienna, Austria; ^2^TAmiRNA GmbH, Vienna, Austria; ^3^Austrian Cluster for Tissue Regeneration, Medical University of Vienna, Vienna, Austria; ^4^Christian Doppler Laboratory for Biotechnology of Skin Aging, Department of Biotechnology, Institute of Molecular Biotechnology, BOKU-University of Natural Resources and Life Sciences, Vienna, Austria; ^5^Laboratory of Navigational Redox Lipidomics and Department of Human Pathology, IM Sechenov Moscow State Medical University, Moscow, Russia

**Keywords:** circulating miRNAs, trauma hemorrhagic shock, ER stress, organ damage, multiple organ dysfunction

## Abstract

Circulating microRNAs (miRNA) alterations have been reported in severe trauma patients but the pathophysiological relevance of these changes is still unclear. miRNAs are critical biologic regulators of pathological events such as hypoxia and inflammation, which are known to induce endoplasmic reticulum (ER) stress. ER stress is emerging as an important process contributing to the development of single and/or multiple organ dysfunction after trauma hemorrhagic shock (THS) accompanied by impaired tissue microcirculation and inflammation. Here, we aim to bring new insights into the involvement of miRNAs associated with ER stress in THS. THS was induced in rats by a median laparotomy and blood withdrawal until mean arterial pressure (MAP) dropped to 30-35 mmHg followed by a restrictive (40 min) and full reperfusion (60 min) with Ringer's solution. Tunicamycin was used to induce ER stress. Blood samples were collected 24 h after THS for the determination of pathological changes in the blood (PCB) and circulating miRNAs. Plasma levels of circulating miRNAs were compared between THS, tunicamycin, and sham groups and correlated to biomarkers of PCB. MiRNA profile of THS animals showed that 40 out of 91 (44%) miRNAs were significantly upregulated compared to sham (*p* < 0.01). The data showed a very strong correlation between liver injury and miR−122-5p (*r* = 0.91, *p* < 0.00001). MiR-638, miR−135a-5p, miR−135b-5p, miR-668-3p, miR-204-5p, miR−146a-5p, miR−200a-3p, miR−17-5p, miR−30a-5p, and miR−214-3p were found positively correlated with lactate (*r* > 0.7, *p* < 0.05), and negatively with base excess (*r* ≤ 0.8, *p* < 0.05) and bicarbonate (*r* ≤ 0.8, *p* < 0.05), which are clinical parameters that reflected the shock severity. Tunicamycin significantly modified the microRNA profile of the animals, 33 out of 91 miRNAs were found differentially expressed. In addition, principal component analysis revealed that THS and tunicamycin induced similar changes in plasma miRNA patterns. Strikingly, the data showed that 15 (25.9%) miRNAs were regulated by both THS and tunicamycin (*p* < 0.01). This included miR−122-5p, a liver-specific microRNA, but also miR−17-5p and miR-125b-5p which are miRNAs remarkably involved in unfolded protein response (UPR)-mediating pro-survival signaling (IRE1α). Since miRNAs associated with ER stress are clearly correlated with THS, our data strongly suggest that interaction between miRNAs and ER stress is an important pathologic event occurring during THS. Overall, we consider that the miRNA profile developed in this study can provide a rationale for the development of bench-to-bedside strategies that target miRNAs in critical care diseases or be used as biomarkers in the prognosis of trauma patients.

## Introduction

Trauma hemorrhagic shock (THS) is a leading cause of death worldwide (1). THS is a form of hypovolemic shock in which blood loss leads to impaired oxygen delivery and later on to inflammation and/or impaired immune response (1–4). The most dramatic consequence of THS is the so-called multiple organ dysfunction syndrome (MODS) (5–7). Cellular, tissue, and vascular alterations play a central role in the pathophysiology of organ failure but yet the death of patients upon MODS has no clear rational explanation. Biological consequences resulting from THS as hypoxia and inflammation are consistent factors required to trigger endoplasmic reticulum (ER) stress (8, 9). Activation of ER stress upon trauma hemorrhage was previously reported in preclinical models (10–12). We showed that the first signs of ER stress are detectable 40 min after reperfusion and persisted for up to 18 h after THS (11). Similarly, THS with sustained hypotension (without fluid resuscitation) simultaneously impacted mitochondrial function and ER stress response (12).

ER stress is known to trigger the unfolded protein response (UPR), an evolutionary conserved adaptive response that aims to resolve ER stress (adaptation, autophagy) or induce cell death if ER stress cannot be solved (8, 13). THS is based on a primary induction of shock by bleeding upon which an induction of acute inflammation occurs as a secondary aseptic inflammation which is mainly induced by damage-associated molecular patterns (DAMPs) (14), but also by gut-derived bacterial translocation (15). Recently, it was anticipated that ER stress is linked to sterile inflammation due to the release of misfolded (unfolded) proteins which may activate immune responses through the release of DAMPs into the circulation (16). And, in fact, it was previously shown that severe ER stress causes the release of extracellular vesicles carrying proinflammatory DAMPs molecules (17). Exactly how ER stress would lead to the release of DAMPs is still not clear, but it is assumed that autophagic vesicles create amphisomes that fuse to the plasma membrane to free the excess of misfolded proteins in an effort to support proteostasis recovery (16). Experimentally (*in vitro* and *in vivo*) ER stress is broadly induce by tunicamycin, thapsigargin, and brefeldin A (18). Although those chemicals target distinct ER machinery, all lead to ER protein misfolding. In this study, activation of ER stress by tunicamycin treatment was chosen since it is the most prototypical agent used to induce pharmacologic ER stress and UPR. Tunicamycin is an inhibitor of protein N-linked glycosylation impairing newly synthesized proteins (19), leading to the disruption of their folding and to the accumulation of unfolded proteins in the ER.

Recently, extracellular vesicles and particles carrying microRNAs (miRNAs) have been identified as new important regulators of intercellular communication and signaling. miRNAs are fine-tune regulators of multiple processes such as gene and protein expression (20, 21), but also of cellular stress responses such as ER stress (22–24). While we have a substantial understanding of mechanisms that control gene expression and protein translation, the involvement of miRNAs in UPR has only been investigated in the past few years (25). MiRNAs circulating in the bloodstream have also been reported to serve as an important diagnostic tool since they are accessible, stable, biologically relevant, and can, therefore, function as clinical biomarkers (26–29). In this scenario, miRNAs released into the blood offer an advantage over miRNAs expressed in tissues since in human studies fresh biopsy material with yielding high-quality RNA is not always possible to obtain (30). In 2014, the group of Stephen L. Barnes identified circulating miRNAs differentially expressed in hemorrhagic shock patients (31). Thereby, direct and indirect correlations within the differentially expressed miRNAs and genes involved in the toll-like receptors (TLRs) signaling pathways were described (31). TLR4 is known to be a prerequisite for activation of a systemic inflammatory response associated with hepatocellular injury after hemorrhagic shock and resuscitation (32). In general, attenuation of inflammatory response ameliorates organ damage (33). Hemorrhagic shock-activated neutrophils augment TLR4 signaling in lung injury (34). Induction of IL-6, a cytokine activated by TLR4, has been reported to ameliorate liver injury in THS (35), and TNF-α release is suppressed immediately after hemorrhage and resuscitation (15). MiRNAs are also recognized as important regulators of the inflammatory response (36–38) but the relationship between miRNAs, inflammation, and tissue damage is unclear and profiling of miRNA patterns upon THS is very scarce. Knowledge on such miRNA patterns, however, might provide valuable information for better diagnosis and a rationale for the development of bench-to-bedside strategies by e.g. targeting miRNAs. We here assumed that miRNAs are key regulators of cellular processes such as ER stress that occurs after THS. Consequently, the aim of this study was to bring new insights into the involvement of circulating miRNAs associated with organ damage and ER stress in THS and its relevance in the development of multiple organ dysfunction. Indeed, we here identified circulating miRNAs in a preclinical model of THS and correlated them with pathological changes in the blood (PCB). In addition, we compared the miRNA profile obtained upon THS with miRNAs induced by tunicamycin in order to clarify whether or not ER stress mediates the release of similar miRNAs as THS does.

## Materials and Methods

### Ethics

The animal protocol was reviewed by the board of the city government of Vienna, Austria and approved for all experimental procedures (GZ: 593334/2016/13). All experiments were conducted in a manner that discomfort, pain, distress and suffering was avoided or minimized and in accordance with the Guide for the Care and Use of Laboratory Animals as defined by the National Institutes of Health. 3Rs concept: All experiments were planned carefully to ensure a sufficient number of animals to enable statistical analysis but not more than that. Experiments were combined to reduce the number of animals. All animal experiments were performed under deep anesthesia (during surgical intervention) and pain killers in pre and post-operative stage (refinement).

### Animals

In total, 17 male Sprague-Dawley rats (400-450g, Janvier Labs, France) were kept under standard conditions, with free access to standard laboratory chow diet and water. All animal experiments were randomized.

### Traumatic Hemorrhagic Shock Model

The traumatic hemorrhagic shock model used in this study is a well-established and clinically relevant model that has been used and reported in previous studies (11, 12). The animals were fasted for 12 h before the experiment with free access to water and received 0.05 mg/kg buprenorphine (Bupaq, Richter Pharma AG, Vienna) subcutaneous at least 1 h before experiment started. Under isoflurane inhalation (Forane, AbbVie, Austria) anesthesia a catheter was placed in the jugular vein. Once the catheterization was completed, the isoflurane anesthesia was terminated and preserved with intravenous continuous drip application of S-ketamine (60 mg/kg/h, Ketanest-S, Pfizer, Austria) followed by an intramuscular application of xylazine (2.5 mg/kg, Rompun, Bayer, Austria). The intravenous application of the S-ketamine permits a well-controlled deep anesthesia (39). Animals were placed on a temperature-controlled surgical board (36–37°C) during operation. A scheme of the experimental procedure is deciped in Supplementary Figure 1A. A catheter for MAP, heart rate, volume therapy, and blood sampling was placed intraoperative in the femoral artery. After cannulation of the jugularis vein and femoral artery, hemodynamic parameters were allowed to stabilize for at least 10 min (baseline—Supplementary Figure 1A). MAP and heart rate were monitored using a PowerLab software system (ADInstruments Ltd., Oxford, UK)—data is displayed in Supplementary Figures 1B,C, respectively. Subsequently, a median laparotomy was performed to mimic tissue trauma (40). The incision remained protected with saline-soaked gauze and sutured after 20 min. The shock phase was initiated by blood withdrawal via the femoral artery catheter. The first 6 mL of blood were manually drained using syringes (baseline sampling). Then 10 mL of blood were withdrawn with a pump (1 ml/min) to reduce the blood pressure (30–35 mmHg) and maintained until reversible decompensation (end of shock—Supplementary Figure 1A). By definition, cardiovascular decompensation is the failure of the cardiovascular system to regulate blood pressure in the face of extreme intravascular volume loss (hypotension), which at this phase can only be prevented by constant fluid infusion. In our experiments, beginning of reversible decompensation was characterized by a rapid drop in MAP from 35 mmHg to around 30 mmHg despite immediate intra-arterial application of small amounts of Ringer's lactate. MAP, heart rate, and blood gas parameters were used to determine the beginning of decompensation, which was quantitatively confirmed by pH about 7.25–7.15, base excess between−11 and−14 and lactate >50 mg/dL. The volume of shed blood (including sampling volumes) withdrawn from THS animals was ~45% of the total blood volume, which represents 7% of the rat body weight. A restrictive reperfusion (MAP at 40–50 mmHg) of 40 min was performed to simulate the transport from the local accident to the hospital and it was followed by a full reperfusion phase for 60 min. The infused volume of Ringer's lactate solution during reperfusion was 5 times the shed volume. Blood samples were withdrawn before shock (baseline), at the end of shock, and 24 h after THS for different analysis. Catheters were removed at the end of reperfusion (Supplementary Figure 1A) and wounds were closed. Subsequently, the animals under analgesia were transferred to their cages and observed until 24 h post-THS. The animals were euthanized under deep anesthesia by decapitation with a guillotine to avoid secondary effects since the animal's main organs were saved for future studies. Here, 14 male Sprague-Dawley rats were randomly distributed into two groups: the sham group (*n* = 4) and THS group (*n* = 8). Two animals (one from the sham group and other from THS group) died before the endpoint (24 h) and were therefore excluded from the analysis. One animal died during preparation (sham group) and the other died after THS. The sham group represents the group of animals subjected only to placebo surgery without the trauma procedure and hemorrhage. Only THS group received phosphate buffered saline with 0.67% of DMSO (3 ml).

### ER Stress Induction

Tunicamycin (TUN), an inhibitor of protein N-glycosylation that impairing protein synthesis and triggers accumulation of unfolded proteins in the endoplasmic reticulum was selected to induce ER stress response in animals randomly assigned to tunicamycin group. 0.5 mg/kg of tunicamycin in phosphate buffered saline with 0.67% of DMSO solution was applied intraperitoneal. Blood was collected after 24 h treatment for determination of circulating miRNAs. Tunicamycin group (*n* = 3).

### Blood Gas Analysis

Blood gas parameters as lactate (Lac), base excess (ABEc), and bicarbonate (${\text{HCO}}_{3}^{-}$) were analyzed in heparinized arterial blood samples with a blood gas analyzer ABL800 Flex System (Radiometer Medical A/S, Copenhagen, Denmark) at the end of shock.

### Blood Cell Count

Total white blood cells (WBC), neutrophils (NEU), lymphocytes (LYM), monocytes (MONO) were measured 24 h after onset of shock on EDTA-anti-coagulated whole blood using a CELL-DYN 3700 analyzer (Abbott Park, Illinois, U.S.A.).

### Pathological Changes in the Blood

The levels of lactate dehydrogenase (LDH), creatine kinase (CK), alanine aminotransferase (ALT), aspartate aminotransferase (AST), urea and creatinine were analyzed from fresh frozen plasma samples with automatic analyzer (Cobas c111; Roche Diagnostics, Vienna, Austria). Cardiac troponin I (cTnI) it was measured using an Ultra-Sensitive Rat Cardiac Troponin-I ELISA kit (Life Diagnostics, Inc., Cat. No. CTNI-2-US). To avoid unintended biasing of the results we performed blind analysis of the samples. Measurements were performed by an investigator unaware of the sample group allocation during the experiment and when assessed its outcome. Pathological changes in the blood (PCB) were analyzed by creating scorings for heart injury (cTnI, CK, LDH), liver injury (ALT and AST), kidney injury (urea and creatinine), cell response [WBC and neutrophil to lymphocyte ratio (NLR)], and tissue hypoxia (lactate and ABEc). Each score was calculated as a sum of the parameters and normalized to the mean value of the THS group and expressed in percent (%). The scores are displayed in Table 1.

**Table 1:** Pathological changes in the blood (PCB) analyzed in THS rat model.

**Pathophysiological event**	**Pathological changes in the blood**	**THS (*n* = 8)**	**sham (*n* = 4)**	**Fold change**	***p value***
Heart injury	Cardiac troponin I (ng/mL)	1.150	0.033	34.6	0.1327
	Creatine kinase (U/L)	37499	898	41.8	0.0661
	Lactate dehydrogenase (U/L)	6766	383	17.7	0.0160
	**Scoring** (cTnI + CK + LDH) **%**	**300**	**10.94**	**27.4**	**0.0063**
Liver injury	Alanine aminotransferase (U/L)	688	153	4.5	0.0082
	Aspartate aminotransferase (U/L)	3279	692	4.7	0.0006
	**Scoring** (ALT + AST) **%**	**200**	**43.25**	**4.6**	**0.0005**
Kidney injury	Urea (μmol/L)	99	42	2.3	0.0592
	Creatinine (μmol/L)	58.38	34.48	1.7	0.1512
	**Scoring** (urea + creatinine) **%**	**200**	**101.95**	**2.0**	**0.0859**
Cellular response	White blood cells (K/μl)	9.82	14.85	0.7	0.0273
	Neutrophils (K/μl)	4.36	3.05	1.4	0.0847
	Lymphocytes (K/μl)	5.25	11.11	0.5	0.0390
	Monocytes (K/μl)	0.08	0.21	0.4	0.0584
	Neutrophil to lymphocyte ratio (NLR)	1.07	0.32	3.3	0.0203
	**Scoring** (WBC + NLR) **%**	**200**	**79.17**	**2.5**	**0.0115**
Tissue hypoxia	Lactate (mg/dL)	34.63	4.50	7.7	<0.00001
	Base excess (mmol/L)	−10.20	−1.03	10.0	<0.00001
	Bicarbonate (mmol/L)	16.54	22.93	0.7	<0.00001
	**Scoring** (lactate + base excess) **%**	**200**	**23.05**	**8.7**	** <0.00001**

*Clinical parameters of animals subjected to THS and sham animals (control group) were collected 24 h upon THS. Biochemical blood parameters as lactate, base excess, and bicarbonate (${\text{HCO}}_{3^{-}}$) were determined at the end of shock. The mean values of THS and sham group, fold changes, and p value are displayed. Scorings (bold values) are described in the methods section. Statistical analysis was performed using Welch's t-test*.

### RNA Extraction and RT-PCR

Ribonucleic acid (RNA) isolation was performed from 200 μl of rat plasma as described previously (41) using the miRNeasy Mini Kit (Qiagen, Germany) together with glycogen to enhance precipitation. The miRCURY RNA Spike-Ins (Qiagen, Germany) were added to the lysis buffer Qiazol prior to RNA isolation. Total RNA was eluted in 30 μl nuclease-free water and frozen at−80°C until further analysis. Reverse transcription was performed using the miRCURY RT Kit (Qiagen, Germany) with 2 μl total RNA input. RT was performed at 42°C for 60 min followed by heat inactivation at 95°C for 2 min. Quantitative polymerase chain reaction (qPCR) was performed on a Roche LightCycler LC480 II with 45 amplification cycles (95°C for 10 s, 60°C for 60 s) followed by melting curve analysis. Cq-Values were calculated using the 2nd derivative maximum method. Data normalization: Equal biofluid volumes were used throughout the analysis. Homogeneous efficiency of all steps in the workflow was confirmed using spike-in controls. RNA spike-in control was used for normalization to adjust for analytical noise using the equation: Cq = Cq^(UniSp4)^ – Cq^(miRNA)^.

### RT-qPCR Quality Control

Three types of Spike-In controls were used for monitoring the analytical variability of the workflow assess data quality (Supplementary Figure 2). First, a commercial (Qiagen, Germany) non-mammalian RNA spike-In (UniSp 4) was added to each sample prior to RNA extraction. RNA spike-Ins reflect the overall technical variance present in the raw data. Secondly, cel-miR-39, which is a microRNA that is found in C. elegans but not in human or other mammalian species was added to total RNA samples prior to reverse transcription. Cel-miR-39 reflects the variance introduced during reverse transcription and qPCR, and can indicate the presence of enzyme inhibitors. Third, UniSp3 was used, which is a mix of qPCR primers and template that is present on each qPCR plate and used to determine the technical variance of the qPCR reaction.

### miRNAs Profiling and Analysis

We have selected and analyzed 91 miRNAs (Supplementary Table 1) associated either with organ damage or ER stress based on existing literature using miRBase database (http://www.mirbase.org/). For the Venn diagram we used Venny 2.1 (https://bioinfogp.cnb.csic.es/tools/venny/index.html). Principal component analysis (PCA) was carried out with the XLSTAT software (Microsoft).

### Statistics

Statistical analysis was carried out with SPSS15 (Statistical Package for the Social Sciences, version 15.0) and the free software environment for statistical computing and graphics, “R.” Kolmogorov–Smirnov test was used to test the distribution of the data. Column analysis was performed using Welch's *t*-test (type 3 *t* test in the EXEL) for Unequal Variances. Volcano plots and Spearman's correlation coefficients were performed R software system using the *EnhancedVolcano* and *corrplot* packages. Significant differences were considered at *p* < 0.01 for the miRNAs analysis, while the criteria for significant correlations was *p* < 0.05.

## Results

### Tissue Hypoxia, Inflammation, and Organ Damage in THS

A total of 14 animals were used in the THS experiment, of which *n* = 4 rats were randomized to the sham group and *n* = 8 rats to THS group, 2 animals were excluded since they did not survive to the endpoint (24 h). Changes in MAP and heart rate of sham and THS animals are shown in Supplementary Figures 1B,C, respectively at baseline, end of shock and, end of reperfusion. For the analysis of PCB induced by THS compared to sham animals, we analyzed the scorings created for the liver, heart, and kidney cumulative damage (Table 1). Alterations related to cellular response and tissue hypoxia are also considered in Table 1. Neutrophils, which are among the first cells to reach the site of injury had fold change of 1.4 (THS = 46.83% and sham = 21.95%) 24 h after onset of shock. The overall inflammatory response scoring (WBC and NLR) was elevated by 2.5-fold (*p* < 0.05) in THS. The data showed a significant increase of lactate in THS animals after shock phase which is accompanied by significant changes in base excess and bicarbonate levels (*p* < 0.0001). In general, all parameters had a strong trend to increase but heart injury scoring was the most elevated by 27.4-fold in THS (*p* < 0.01). Liver and kidney injury were elevated by 4.6 and 2.0-fold, respectively.

### miRNA Profile of THS Rats

**Figure 1:**
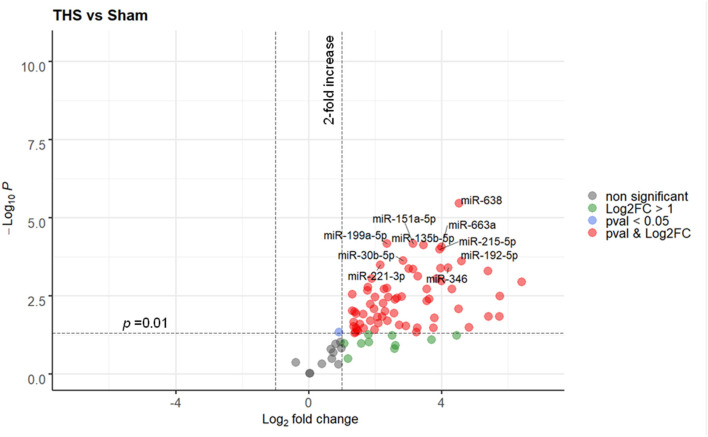
Volcano plot depicting the Log_2_-transformed fold change (LogFC, x-axis) of circulating miRNAs in THS compared to controls (sham) in relation to their *p* value (−Log10 (pval), y-axis). In total 90 miRNAs were included in the analysis. The 10 most significantly regulated miRNAs (lowest pval) by THS are labeled. Sham group (*n* = 4 animals); THS group (*n* = 8 animals).

Ninety one miRNAs associated to organ damage and ER stress were selected based on existing literature (Supplementary Table 1) and determined their relative expression in plasma samples of THS and sham (control) animals by RT-qPCR. Spike-in quality controls were used to monitor analytical variability and sample quality (Supplementary Figure 2). Overall, 40 out of 91 miRNAs were significantly elevated (*p* < 0.01) in the animals subjected to THS compared to the sham group, while none were decreased (Figure 1). The full list of the miRNAs induced by THS with a *p* < 0.01 is displayed in Supplementary Figure 3. The data showed that miR-638, miR-199a-5p, miR-151a-5p, miR-135b-5p, miR-663a, miR-215-5p, miR-30b-5p, miR-192-5p, miR-221-3p, and miR-346 are the 10 most significantly regulated miRNAs in THS plasma (Figure 1). Additionally, several miRNAs such as miR-122-5p (log_2_ fold change = 6.41), miR-211-5p (log_2_ fold change = 5.76), and miR-135a-5p (log_2_ fold change = 5.40) occurred in the blood in particularly high concentrations.

### Correlations Between PCB and Circulating miRNAs in THS

**Figure 2:**
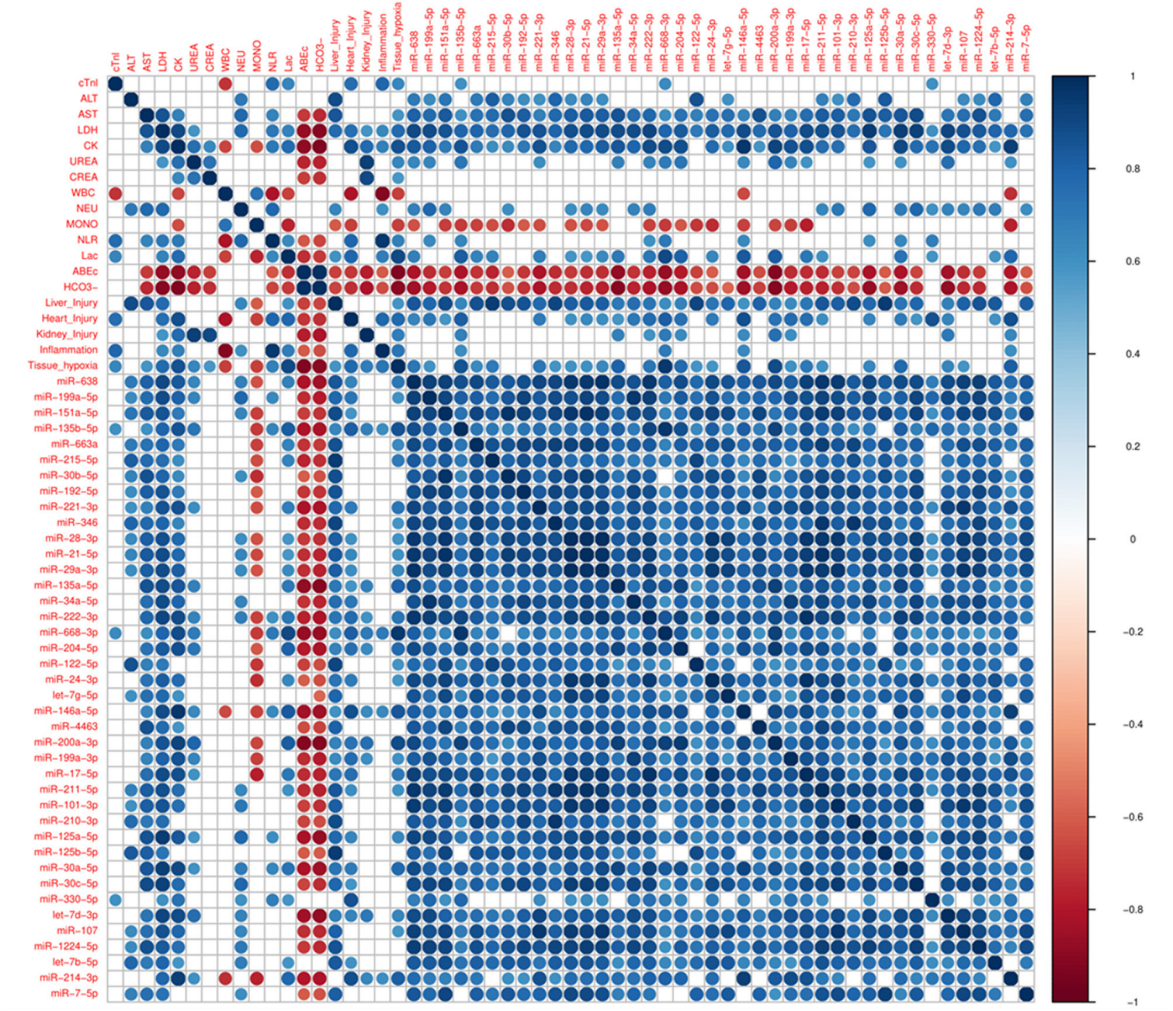
Corrplot with Spearman's correlations between pathological changes in the blood (PCB) and circulating miRNAs induced by THS with *p* < 0.01 (*n* = 40 miRNAs). All significant (*p* < 0.05) correlations are indicated as circle. Positive correlations are displayed in blue and negative correlations in red color. Color intensity and the size of the circle are proportional to the correlation coefficients (r).

Next, it was explored whether the changes in miRNAs in THS are coincident with PCB, which are used in clinical practice. 40 miRNAs induced by THS were selected for performing the correlation analysis with the PCB (*p* < 0.01). Figure 2 displays all significant Spearman's correlations between miRNAs and PCB (*p* < 0.05). The coefficients (r) between PCB and circulating miRNAs are indicated in Supplementary Figure 4. In general, all the miRNAs induced by THS are directly correlated to each other (blue circles). The data did not show an organ-specific miRNA profile but most of the miRNAs were significantly correlated with liver injury (38 out of 40), followed by heart injury and hypoxia (26 out of 40), kidney injury (10 out of 40) and inflammation (4 out of 40) (*p* < 0.05). The data showed a strong correlation between liver injury scoring and miR−122–5p (*r* = 0.91, *p* < 0.0001). Furthermore, miR−135b−5p, miR-668-3p, and miR−146a−5p were found to be associated with all the 5 scorings (liver injury, heart injury, kidney injury, and inflammation, and tissue hypoxia). Additionally, miR−135b−5p, miR−668–3p, miR−146a−5p, and miR−214–3p are the only miRNAs correlated with activation of immune cells (WBC + NLR). Some PCBs are also strongly correlated with each other. For example, heart injury with inflammation (*r* = 0.78, *p* < 0.01), tissue hypoxia (*r* = 0.78, *p* < 0.01), and kidney injury correlates with tissue hypoxia (*r* = 0.69, *p* < 0.05). Base excess and bicarbonate (${\text{HCO}}_{3}^{-}$), clinical parameters that reflect shock severity, are negatively correlated with all miRNAs (red circles). Base excess and bicarbonate are also inversely correlated with AST, LDH, CK, urea, creatinine, and lactate. Here, miR-638, miR−135b, miR−135a, miR-668, miR-204, miR−146a, miR−200a, miR−17, miR−30a, and miR−214 were found positively correlated with lactate (*r* > 0.7, *p* < 0.05), negatively with base excess (*r* ≤ 0.8, *p* < 0.05) and bicarbonate (*r* < −0.8, *p* < 0.05).

### miRNA Profile of Tunicamycin in Rats

**Figure 3:**
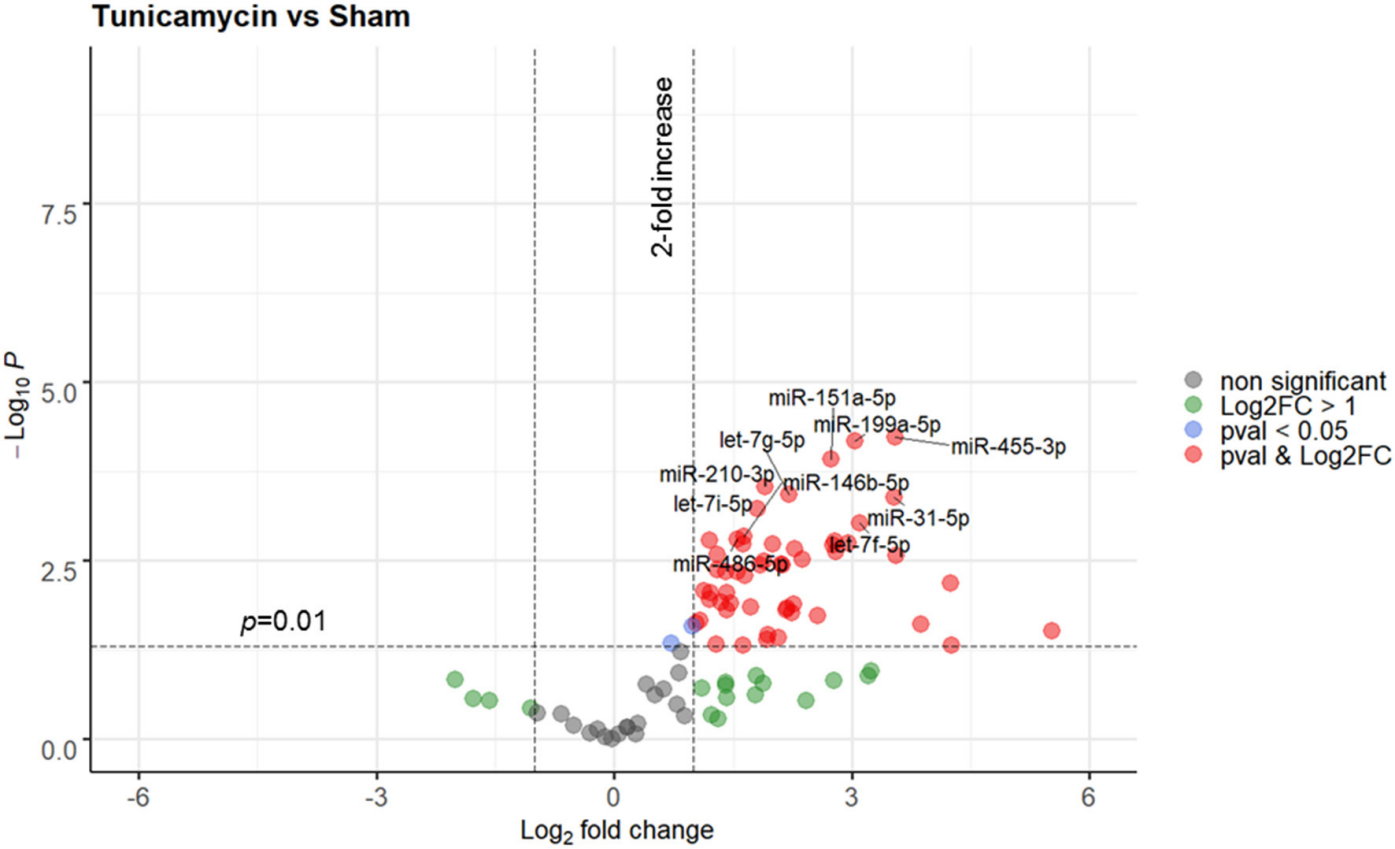
Volcano plot depicting the Log_2_-transformed fold change (LogFC, x-axis) of circulating miRNAs induced by tunicamycin compared to controls (sham) in relation to their *p* value [−Log10 (pval), y-axis]. The 10 most significantly regulated miRNAs (lowest pval) by tunicamycin are labeled. Sham group (*n* = 4 animals); Tunicamycin group (*n* = 3 animals).

In the next step, ER stress response was triggered in rats using a strong and well-known inducer of ER stress, tunicamycin. A total of 3 animals were used in this experiment, all animals were included in the analysis. No animal died due to treatment with tunicamycin. Similar to THS group (24 h), the levels of circulating miRNAs in animals treated with tunicamycin (24 h) were strong upregulated compared to sham group. Overall, 33 out of 91 miRNAs were significantly upregulated (*p* < 0.01), while again, none were decreased. A full list of the miRNAs expressed in response to tunicamycin with a *p* < 0.01 is displayed in Supplementary Figure 5. A detailed analysis showed that miR-455-3p, miR-199a-5p, miR-151a-5p, miR-210-3p, let-7g-5p, miR-31-5p, let-7i-5p, let-7f-5p, miR-146b-5p, and miR-486-5 were the 10 most upregulated miRNAs by tunicamycin in our experimental model (Figure 3).

### A Common miRNA Signature in THS and Upon ER Stress Stimulation With Tunicamycin

**Figure 4:**
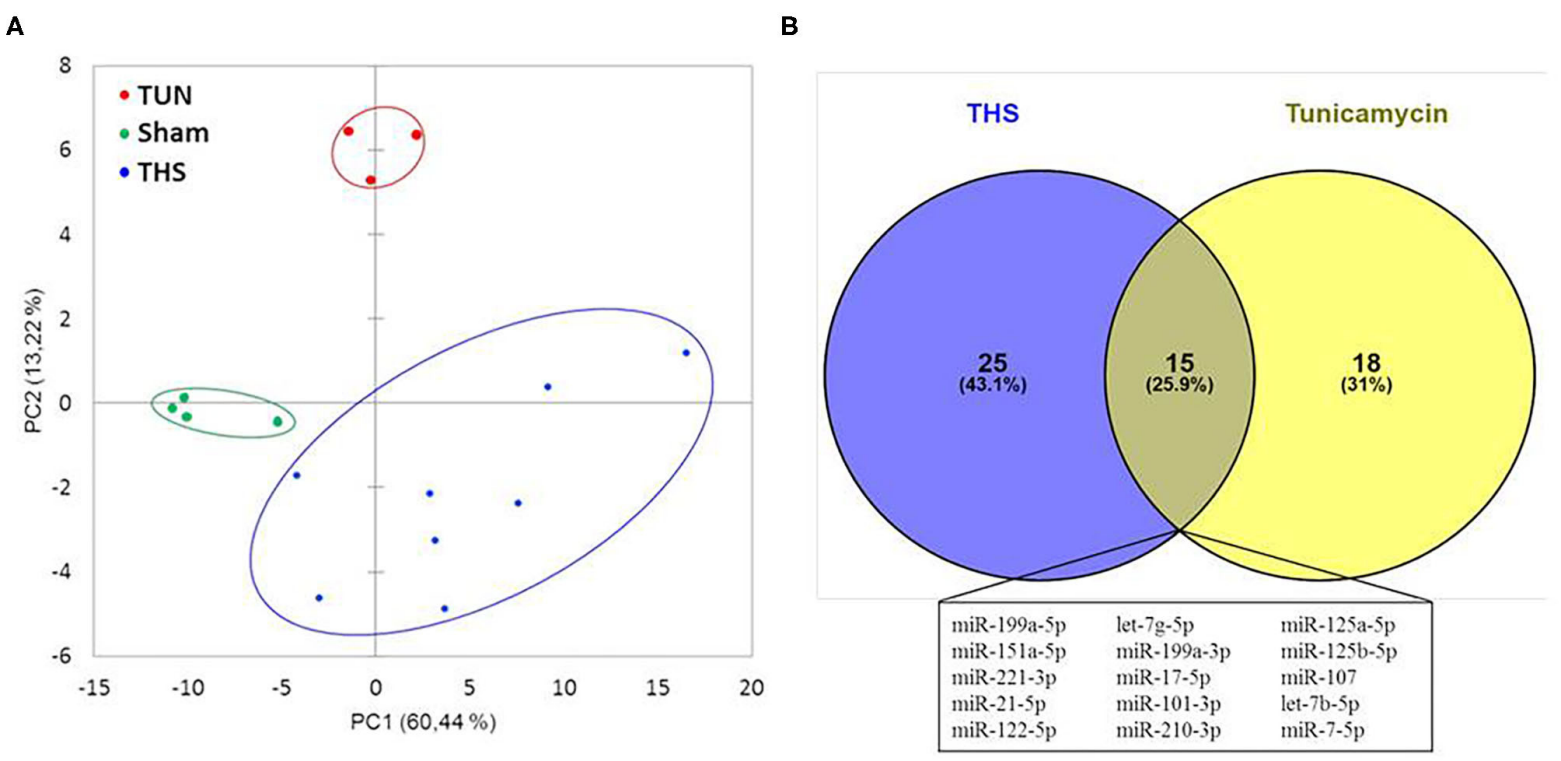
**(A)** Principal component analysis of miRNAs related to organ injury and ER stress; THS group (*n* = 8); sham group (*n* = 4); tunicamycin (TUN) group (*n* = 3). **(B)** Venn diagram with miRNAs significantly regulated in THS and upon tunicamycin treatment (*p* < 0.01). Blue: 25 miRNAs involved exclusively in THS. Yellow: 18 miRNAs exclusively induced by tunicamycin. Central area: 15 common miRNAs expressed by THS and tunicamycin.

Circulating miRNAs released in THS animals were compared to tunicamycin group in relation to sham group (control). The principal component analysis revealed that THS and tunicamycin treatment caused similar miRNA changes compared to sham group (Figure 4A). THS and tunicamycin showed a different miRNA profile as well, but more similar to each other than to sham since both groups overlap on PC1 (x-axis, explained 60% variability) but not on PC2 (y-axis, explained 13% variability). Next, a direct comparison of significantly upregulated miRNAs in THS and tunicamycin with a *p* < 0.01 was performed and displayed in Figure 4B. The Venn diagram showed that 25 miRNAs were exclusively induced by THS and 18 miRNAs were exclusively induced by tunicamycin treatment. This analysis also identified 15 common miRNAs induced by both, THS and ER stress: miR-199a-5p, miR-151a-5p, miR-221-3p, miR-21-5p, miR-122-5p, let-7g-5p, miR-199a-3p, miR-17-5p, miR-101-3p, miR-210-3p, miR-125a-5p, miR-125b-5p, miR-107, let-7b-5p, and miR-7-5p. In other words, THS and tunicamycin had an overlap of 25.9% of miRNAs analyzed in our study.

## Discussion

MiRNA transcription has previously been reported to be altered in severe trauma patients (31, 42, 43). The role of ER stress following THS is not fully understood but since the ER is responsible for main liver functions as protein synthesis, lipid metabolism, etc., we assumed that ER stress can be a major reason for liver dysfunction contributing to multiple organ failure in THS. However, the relationship between circulating miRNAs, activation of ER stress, and organ damage is very poorly explored. Here, we show that circulating miRNAs upon THS can be used to decipher the role of ER stress in the development of single and/or multiple organ dysfunction. In this study, we determined the major parameters of oxygen deficit during the shock phase, subsequent markers of organ damage, and inflammatory response 24 h after shock. The experimental model simulated a typical situation of clinical trauma. Base excess and bicarbonate were significantly decreased during the shock phase reflecting tissue hypoxia. In line with related literature (44), activation of the innate inflammatory response was characterized by elevated levels of neutrophils and a drop in the absolute number of lymphocytes.

In this model, we identified 40 miRNAs differently regulated upon THS (all upregulated). MiR-638 was the most significantly upregulated miRNA induced by THS in our study. Overexpression of miR-638 was found to attenuate the effects of hypoxia/reoxygenation in human cardiomyocytes (45). Previously, it was anticipated that in general miRNAs act as pro-adaptive molecules during ER stress and they can be positively and negatively regulated by the UPR (25). Indeed, we found that the most upregulated miRNAs by THS in our study are likely not only involved in organ damage as liver injury (miR-151a-5p), brain injury (miR-135b-5p), and cardiac injury (miR-199a-5p) but there are also miRNAs in this group which are reported to be involved in processes related to ER stress (miR-663a, miR-215-5p) and UPR activities such as cell survival (miR-346) (references are indicated in Supplementary Table 1). Considering the marked release of the miRNAs upon THS, we also assume that the miRNA profile obtained may not only reflect regulation of ER stress and organ injury but also possible cellular disruption followed by a passive release of miRNA into the bloodstream, which was previously observed during drug-induced organ damage (46, 47). Furthermore, the upregulation of miR-24-3p (log_2_ fold change = 1.79) observed here in the animals subjected to THS (*p* < 0.001) concurs with the overexpression of miR-24-3p reported in trauma patients associated with trauma-induced coagulopathy by preventing the production of coagulation Factor X (42). MiR-24 was also found significantly deregulated under UPR circumstances in H9c2 rat cardiomyoblasts (48).

Based on correlation analysis between miRNAs and PCB, we found that miRNAs are associated with injury of different organs, most importantly liver injury, since 38 out of 40 upregulated miRNAs were correlated to the liver injury score. In addition, we found a strong and direct correlation between liver injury and miR−122-5p (*r* = 0.91, *p* < 0.0001), which confirms previous observations by others (49, 50), and which can be explained not only by passive release but also by the augmentation of miR-122 in the liver. MiR-122 was also found to play a role in the hypoxia responses that regulate glucose and energy metabolism (51). In addition, it was recently reported that ER stress impacts on miR-122 promoter activity (52). We found miR-638, miR−135a-5p, miR−135b-5p, miR-668-3p, miR-204-5p, miR−146a-5p, miR−200a-3p, miR−17-5p, miR−30a-5p, and miR−214-3p strongly correlated with lactate, base excess, and bicarbonate. Base excess is reflected in bicarbonate consumption, which results in acidosis (53). In fact, miR-638, miR−135a, miR-668 were previously associated with hypoxic injury (references are indicated in Supplementary Table 1). Thus, our data suggest that these miRNAs could also be explored as markers of tissue hypoxia subsequently to THS. We observed that only a few miRNAs (miR−135b−5p, miR−668–3p, miR−146a−5p, and miR−214–3p) correlated with cellular response scoring (inflammatory response), out of which miR-146a-5p is considered an “inflammamiR” (54, 55). Additionally, in our study, we found several miRNAs strongly increased in the plasma in response to tunicamycin. Of note, tunicamycin may not fully mimic the ER stress response induced by THS, but it's known that the liver is one of the main organs affected by tunicamycin treatment (56). Additionally, since liver dysfunction is also a driver of multiple organ failure in THS, we consider that the choice of tunicamycin as an ER stress model in this study in comparison to the THS model is a suitable approach. And, indeed, miRNAs such as miR-17-5p and miR-125b, which we found to be significantly upregulated in THS and in tunicamycin groups, are associated with ER stress and UPR. MiR-17 and miR-125b have a role in adaptive UPR signaling by regulating IRE1α (57). Notably, it was found that sustained activation of IRE1α reduced levels of miR-17, miR-34a, miR-96, and miR-125b which are miRNAs that usually inhibit caspase-2 (57). PCA analysis revealed that THS and tunicamycin treatment elicit similar changes in plasma miRNA levels compared to control animals. Indeed, we found a coincidence of 25.9% miRNAs in THS and upon ER stress stimulation. 15 miRNAs (miR-199a-5p, miR-151a-5p, miR-221-3p, miR-21-5p, miR-122-5p, let-7g-5p, miR-199a-3p, miR-17-5p, miR-101-3p, miR-210-3p, miR-125a-5p, miR-125b-5p, miR-107, let-7b-5p, and miR-7-5p) were significantly induced by both THS and tunicamycin. In this list, we highlight miR-122-5p, miR-17-5p, and miR-125b-5p which we discussed above.

**Figure 5:**
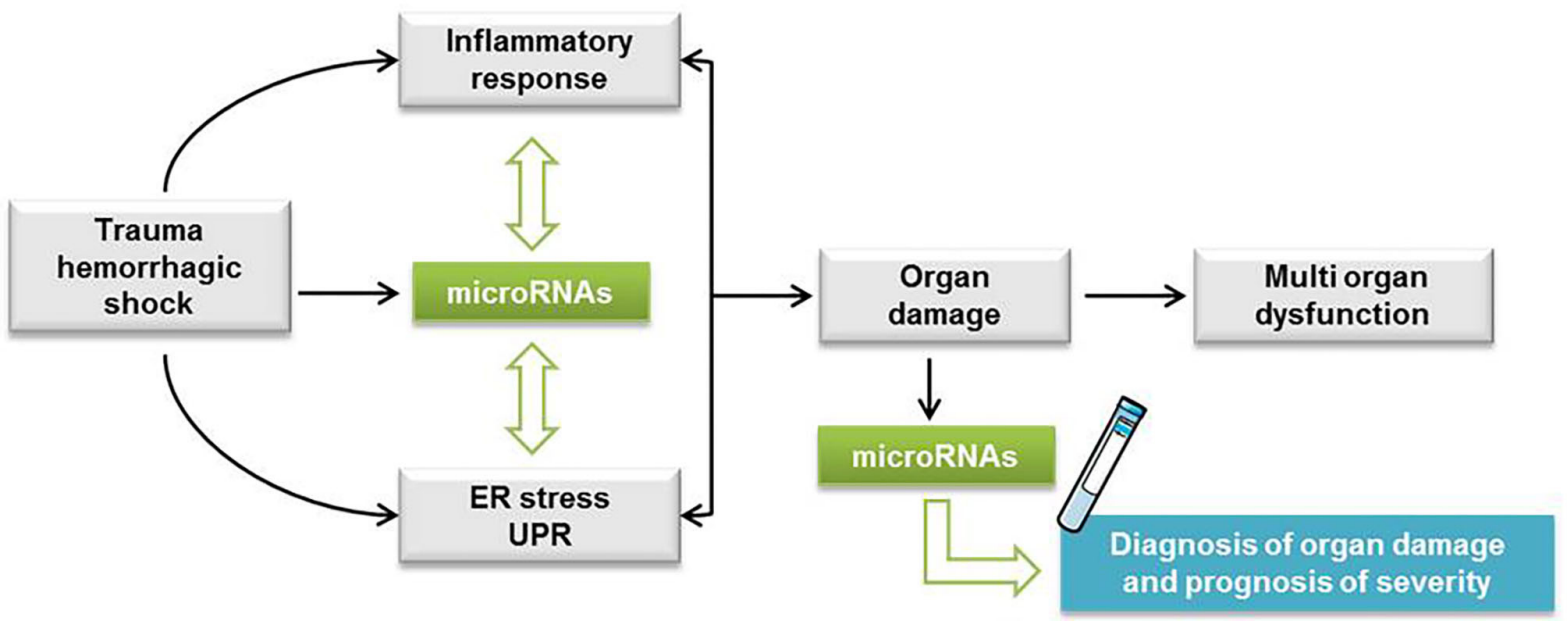
Hypothetic scheme with the interplay between ER stress and miRNAs upon THS. Cellular perturbations resulting from trauma hemorrhagic shock (THS) activate inflammation and endoplasmic reticulum (ER) stress which triggers the unfolded protein response (UPR). At the same time, THS also induces the release of miRNAs in the plasma that fine-tune ER stress/UPR and inflammatory response. The feed-forward loop comprising the activation of inflammation and ER stress is implicated in the development of cell damage followed by single and multiple organ dysfunction.

We propose that our miRNA profile characterization in THS animals can provide a rationale for the development of bench-to-bedside strategies that target miRNAs. For example, miR-34a, which we found induced by THS (*p* < 0.01) and by tunicamycin (*p* < 0.05) was suppressed in a hemorrhagic shock pregnant rat model. The authors found that suppression of miR-34a alleviates organ damage possibly due to the attenuation of oxidative stress (58). Thus, suppression of specific miRNAs can be explored to treat organ damage caused by THS. In addition, since the miRNAs identified were associated with PCB, they can also be explored to improve the diagnostic toolbox for trauma patients. In fact, it was previously found that miR-106a was dysregulated in hemorrhagic shock patients and appeared to be more downregulated in patients who developed an infection (43). Thus, specific alterations on miRNA levels were suggested to serve as a marker for susceptibility to infection in trauma patients (43). Considering our data, we assumed the following mechanism with respect to the interplay between ER stress and miRNAs upon THS. Cellular perturbations resulting from THS activate inflammation and ER stress which triggers the UPR. At the same time, THS also induces the release of miRNAs in the plasma that fine-tune ER stress/UPR and inflammatory response (Figure 5). We recently described a feed-forward loop comprising the activation of inflammation and ER stress in the development of liver damage (59). This cycle can be either beneficial contributing to the restoration of tissue homeostasis or deleterious such as cell and organ dysfunction (59).

Summarized, we show that circulating miRNAs associated with ER stress are increased upon THS, suggesting a relationship between ER stress and the release of miRNAs in the blood. The comparison of the miRNA profiles induced by THS in the blood with specific organ damage markers suggested that miRNAs are predominantly released from the liver. Thus, our data strongly suggest that ER stress is an important pathologic event occurring during THS, but its pathophysiological impact is still unclear. Further studies are required to clarify the pathophysiological role of ER stress upon THS. In addition, as more than half of all hospital deaths associated with THS occur after the first 24 h (60), it is relevant to investigate miRNAs associated with ER stress expressed in the later course of organ failure after trauma. Overall, we consider that the miRNA profile characterized in this study provides a rationale to improve the diagnosis of organ damage and the prognosis of trauma patients (Figure 5).

## Data Availability Statement

The raw data supporting the conclusions of this article will be made available by the authors, without undue reservation.

## Ethics Statement

The animal protocol was reviewed by the board of the city government of Vienna, Austria and approved for all experimental procedures (GZ: 593334/2016/13). All experiments were conducted in a manner that discomfort, pain, distress and suffering was avoided or minimized and in accordance with the Guide for the Care and Use of Laboratory Animals as defined by the National Institute of Health.

## Author Contributions

AL, SB, and AK conceived and designed the study. JJ and CK performed the traumatic hemorrhagic shock model in rats. AL and MJ assisted with the experimental performance. AL, AK, and MH contributed to figure creation. AL, MH, JG, and AK contributed to the interpretation and analysis of data. AL and AK drafted the manuscript. All authors read and approved the final version of the manuscript.

## Conflict of Interest

MH is employed by the company TAmiRNA GmbH, Vienna, Austria, and JG is a co-founder and shareholder of TAmiRNA. The remaining authors declare that the research was conducted in the absence of any commercial or financial relationships that could be construed as a potential conflict of interest.

## Abbreviations

ABEc: base excess
ALT: alanine aminotransferase
AST: aspartate aminotransferase
CK: creatine kinase
CREA: creatinine
cTnI: cardiac troponin I
DAMPs: damage-associated molecular patterns
ER: endoplasmic reticulum
${\text{HCO}}_{3}^{-}$: bicarbonate
IRE1α: inositol-requiring enzyme 1 α
Lac: lactate
LDH: lactate dehydrogenase
LYM: lymphocyte
MAP: mean arterial pressure
miRNA: microRNA
MODS: multiple organ dysfunction syndrome
MONO: monocytes
NEU: neutrophils
NLR: neutrophil to lymphocyte ratio
PCB: pathological changes in the blood
qPCR: quantitative polymerase chain reaction
RNA: ribonucleic acid
THS: trauma hemorrhagic shock
TUN: tunicamycin
UPR: unfolded protein response
WBC: white blood cells.
