# Supplementary material for: Circulating miRNAs Associated With ER Stress and Organ Damage in a Preclinical Model of Trauma Hemorrhagic Shock
Source: Front Med (Lausanne). 2020 Sep 24;7:568096. doi: 10.3389/fmed.2020.568096 (PMC7542230; doi:10.3389/fmed.2020.568096)
Supplement: Supplementary file 1 [file Data_Sheet_1.docx]

Supplementary Material

| 1A  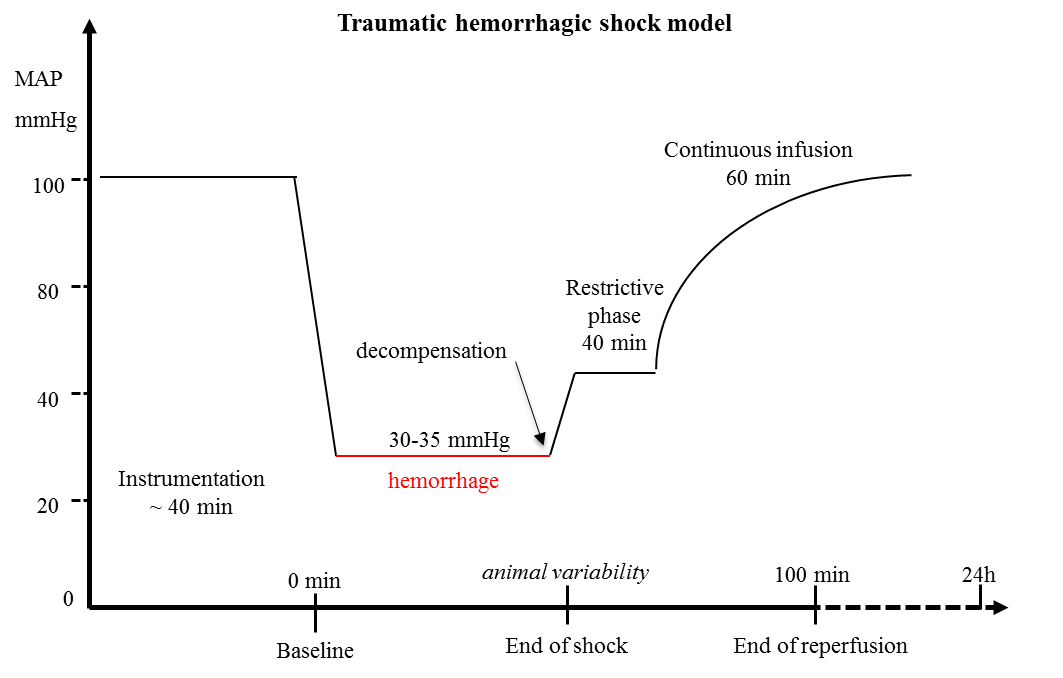 |
| --- |
| 1B   |
| 1C   |

**FIGURE 1|** **A**, Scheme of the THS model applied in rats indicating the three main phases of the model: a controlled hemorrhage until a MAP 30-35 mmHg, a restrictive phase with a partial fluid resuscitation (MAP 45-50 mmHg) and lastly a full resuscitation phase of 60 min (end of reperfusion). Ringer’s lactate was used for the restoration of circulation. The severity of shock was controlled by blood parameters as lactate and base excess. Blood samples were collected at baseline, end of shock, and 24h post-THS for different analyses. **B,** Mean arterial blood pressure (MAP) and **C,** heart rate of sham and THS animals at baseline, end of shock and, end of reperfusion. Sham group (n = 4 animals); THS group (n = 8 animals).

**FIGURE 2|** RT-qPCR quality control. Three types of Spike-In controls where added at the level of RNA extraction (UniSp4), reverse transcription (cel-miR-39), and PCR analysis (UniSp3). Cq-values for each spike-in across all samples are shown. The low variability indicates homogenous RNA extraction efficiency and the absence of RT-qPCR inhibition across the entire set of samples.

**TABLE 1|** Selected miRNAs to this study related to organ injury and ER stress based on existing literature using miRBase database (http://www.mirbase.org/).

| **miRNA** | **Relevance** | **Reference** |
| --- | --- | --- |
| let-7a | brain injury | Song Yang, Shengli Li et al. (2018) Cell Death Dis |
| let-7b-5p | liver injury | Takumi Kagawa, Tsuyoshi Yokoi et al. (2018) Toxicol. Sci. |
| let-7d-3p | cardiac injury | Lee Lee Wong, Peipei Wang et al. (2019) Int J Mol Sci |
| let-7f-5p | endocrine disruption | Shen, Geng-Yang et al. (2019) International journal of biological sciences |
| let-7g | hemorrhagic shock | Juliann G Kiang, Barbara Knollmann-Ritschel et al. (2017) PLoS ONE |
| let-7i-5p | brain injury | Valentina Di Pietro, Antonio Belli et al. (2018) Front Mol Neurosci |
| miR-1 | hypoxia | Yan Lei et al. (2019) Life Sci. |
| miR-101 | liver injury | Hu Song, Zhongyang Shen et al. (2019) Int. J. Mol. Med. |
| miR-107 | brain injury | Valentina Di Pietro, Antonio Belli et al. (2018) Front Mol Neurosci |
| miR-122 | liver injury | Shifeng H, Danni W, Pu C, Ping Y, Ju C, et al. (2013) PLOS ONE |
| miR-1224 | liver injury | Wenting Cheng, Danmei Zhao et al. (2019) J. Cell. Biochem. |
| miR-124 | neuroinjury  hypoxia | Anyong Yu, Zhao Yang et al. (2017) Immunol. Lett.; Liu L, Zang J, Chen X, et al. (2016) Am J Physiol Heart Circ |
| miR-125a-5p | hypoxic injury | Lin Qu, Yang Cao et al. (2018) |
| miR-125b-5p | hypoxic injury  ER Stress | John-Paul Upton et al. (2012) Science; Zuotian Huang, Zhongjun Wu et al. (2019) Biosci. Biotechnol. Biochem. |
| miR-126-3p | vascular injury | Feinberg MW. (2014) J Clin Invest. |
| miR-128 | hypoxic injury | Hua Fang, Jian-Ping Zhang et al. (2019) Biomed. Pharmacother. |
| miR-132 | brain injury | Bing Xu, Jiu-Lin Du et al. (2017) Cell Res. |
| miR-133a | cardiac injury  ER stress | Ren L, Wang Q, Chen Y, Ma Y, Wang D (2019) Pharmacology |
| miR-133b | cardiac injury  ER stress | Danielle E Read et al. (2014) Cell & Bioscience; Hardip Sandhu, Helen Maddock et al. (2017) Eur. J. Pharmacol. |
| miR-134 | hypoxic injury | Sebastian Isac, Ana-Maria Zagrean et al. (2018) Neonatology |
| miR-135a | hypoxic injury | Sheng Wang, Huanzhou Xue et al. (2019) J. Cell. Biochem. |
| miR-135b-5p | brain injury | Valentina Di Pietro, Antonio Belli et al. (2018) Front Mol Neurosci |
| miR-142 | brain injury | Valentina Di Pietro, Antonio Belli et al. (2018) Front Mol Neurosci |
| miR-143 | liver injury | Takumi Kagawa, Tsuyoshi Yokoi et al. (2018) Toxicol. Sci. |
| miR-144 | cardiac injury | Jing Li et al. (2014) & (2018) Basic Res. Cardiol. |
| miR-146a-5p | lung injury, brain injury, liver injury | Jessica L Weaver, Jason W Smith et al. (2016) J. Am. Coll. Surg.; Li Gan et al. (2018) Mediators Inflamm; Lei Chen et al. (2019) Brain Behav Immun. |
| miR-146b-5p | kidney injury | Zhu Y et al., (2016) Stem Cells Transl Med. |
| miR-148 | liver injury | Daofeng Zheng, Zhongjun Wu et al. (2018) Cell. Physiol. Biochem. |
| miR-15 | hemorrhagic shock | Juliann G Kiang, Barbara Knollmann-Ritschel et al. (2017) PLoS ONE |
| miR-150-5p | neuroinjury | Bergman P et al. (2016) Neurol. Neuroimmunol. Neuroinflamm. |
| miR-151 | liver injury | Jin-Lin Cheng, Lan-Juan Li et al. (2018) Hepatol Int |
| miR-155 | sepsis & lung injury  liver injury | Bo Tang et al. (2015) Transpl Int.; Han Y, Li Y, Jiang Y. (2016) Clin Lab |
| mir-17 | brain injury ER stress | John-Paul Upton et al. (2012) Science; Di Chen et al. (2018) J Neuroinflammation. |
| miR-182-5p | hypoxic injury | Xiaohong Zhang et al. (2018) Cell. Physiol. Biochem. |
| miR-185 | cardiac injury | Jin Ock Kim et al. (2016) BMB Rep. |
| miR-191 | liver injury | Wenming Pan, Hui Wang et al. (2019) Cell Death Differ. |
| miR-192-5p | liver injury | Starlinger P et al. (2019) Hepatology. |
| miR-193 | liver injury | Sandra Franco, Cristina Tural et al. (2018) Antiviral Res. |
| miR-199a-3p | brain injury | Xueying Feng et al. (2017) Am J Forensic Med Pathol. |
| miR-199a-5p | cardiac injury, liver injury | B-H Dai, J-M Yang et al. (2013) Cell Death Dis; Yang Zhou, Ying-Bin Xiao et al. (2017) Mol Med Rep; Yang Zhou, Peng Li et al. (2019) J. Physiol. Biochem. |
| miR-200-3p | cardiac injury | Linlin Li, Zhe Wang et al. (2018) Acta Biochim. Biophys. Sin. |
| miR-203 | kidney, brain | Jina Ko, David Issadore et al. (2019) J. Neurotrauma |
| miR-204 | endothelial dysfunction & ER stress | Kassan, M. et al. (2017) Sci Rep 7 |
| miR-206 | ER stress  hypoxia | Danielle E Read et al. (2014) Cell & Bioscience  Fei Kong, Xinglin Duan et al. (2019) Biomed. Pharmacother. |
| miR-208a-3p | cardiac injury | Meng et al. (2015) Asian Pac J Trop Med. |
| miR-208b-3p | hypoxia | Ya-Li Zhou, Rui Li et al. (2018) Biomed. Pharmacother. |
| miR-21 | cardiac, liver, brain injury | Marta B. Afonso et al. (2018) Cell Death Differ.; Yang C et al. (2019) Exp Ther Med; Dai Li, Ping Lei et al. (2019) Med. Sci. Monit. |
| miR-210-3p | lung injury, liver injury | J Zavadil, N Goldberg et al. (2019) Klin Onkol |
| miR-211 | ER stress | Wenna Liang et al. (2019) J Ethnopharmacol. |
| miR-212 | ER stress | Tahir Ali, Iram Murtaza et al. (2019) Arch. Biochem. Biophys. |
| mir-214 | cardiac injury; UPR signalling | Quanlu Duan et al. (2015) J Cell Physiol.; Quanlu Duan et al. (2015) J Transl Med. |
| miR-215 | ER stress | Mian M K Shahzad et al. (2018) PLoS ONE |
| miR-216-5p | pancreas injury | Zhang XX et al. (2017) Am J Med Sci. |
| miR-217 | liver injury | Cheng-Liang Yang, Hui Liu et al. (2019) J. Cell. Physiol. |
| miR-221 | UPR signalling; cardiac injury | Bin Yu, Meifeng Xu et al. (2013) PLoS ONE; Yue Zhou, Peipei Wang et al. (2016) J. Cardiovasc. Pharmacol. Ther. |
| miR-222 | ER stress | Rongyang Dai et al. (2010) Biol Chem. |
| miR-223 | liver injury | Juntaro Matsuzaki, Takahiro Ochiya (2018) J Clin Biochem Nutr |
| miR-24 | ER stress & apoptosis | Chhabra R, Dubey R, Saini N. (2011) RNA Biol.  Danielle E Read et al. (2014) Cell & Bioscience |
| miR-27a | ER stress & apoptosis; severe trauma | Chhabra R, Dubey R, Saini N. (2011) RNA Biol.  Camille G Apple et al. (2020) J Am Coll Surg. |
| miR-27b | brain injury | Wenzhe Xu, Yuguang Liu et al. (2017) Oncotarget |
| miR-28 | hypoxia | Zhiping Chen, Jingjing Meng et al. (2019) Int. J. Biol. Macromol. |
| miR-298 | hypoxic injury | Ling Liu, Guoyu Wang et al. (2019) Pharmazie |
| mir-29a | Hepatoprotection & ER stress & autophagy | Ying-Hsien Huang et al. (2018) Exp Biol Med (Maywood). |
| miR-29b-3p | hypoxia | Yuhua Cai, Yunpeng Li (2019) Cell. Mol. Biol. Lett. |
| miR-30 | brain and cardiac injury | Peng Wang et al. (2015) J Neurosci Res.; Quanlu Duan et al. (2015) J Transl Med. |
| miR-30a-5p | kidney injury | Baker MA et al. (2017) J Am Soc Nephrol. |
| miR-30c-2-3p | UPR signalling | Byrd AE et al. (2012) J Cell Biol. |
| miR-30c-5p | kidney injury | Zou YF et al. (2017) Exp Biol Med (Maywood |
| miR-31-5p | inflammation | Lei Jiang et al. (2018) Biomedicine & Pharmacotherapy |
| miR-320 | cardio protection | Zoltán V Varga, Péter Ferdinandy et al. (2014) Am. J. Physiol. Heart Circ. Physiol.; Ni Yang, Chunfeng Liu et al. (2018) Cell Biochem. Funct. |
| miR-330 | liver injury | Xiang-Li Sun, Shi-Peng Li et al. (2019) J. Cell. Biochem. |
| miR-335 | hypoxic injury | Nan Wu, Ruichao Che et al. (2018) Am J Transl Res |
| miR-340-5p | hypoxic injury | Dong Li, Yan Yu et al. (2019) J. Cell. Biochem. |
| mir-346 | UPR signalling  pro-survival | Junfei Guo et al. (2018) Cancer Lett. 2018 |
| miR-34a-5p | adaptive UPR signalling; cardiac injury | John-Paul Upton et al. (2012) Science; Elena Piegari, Antonella De Angelis et al. (2016) Oncotarget; Clarissa Ruggeri, Yuri D'Alessandra et al. (2018) Dis. Markers |
| miR-367 | liver injury | Fan Yang, Lili Ji et al. (2019) Phytomedicine |
| miR-422a | lung injury | Molina-Pinelo S. et al. (2014) Eur Respir J. |
| miR-423-5p | cardiac injury | Xin Zhu, Xiaolan Lu (2019) J. Cell. Physiol.; Barbara Rizzacasa, Francesca Amati et al. (2019) PLoS ONE |
| miR-4463 | hypoxic injury | Xuemei He, Xiangyu Zhou et al. (2018) Cell. Physiol. Biochem. |
| miR-449b-5p | hypoxic injury | Yong Zhang, Xiaoming Lei et al. (2019) J. Cell. Physiol. |
| miR-455 | lung injury; adaptive UPR signalling | Jiang Hong et al. (2018) Pathol Res Pract. |
| miR-486-5p | cardioprotection | Xiang-Hua Sun, Jie Hui et al. (2019) Thromb. Res. |
| miR-499a-5p | cardioprotection | Xiao Liu, E Wang et al. (2016) Exp Ther Med |
| miR-638 | hypoxic injury | P Zhao, Z-H Li et al. (2018) Eur Rev Med Pharmacol Sci |
| miR-663 | ER stress | Gargalovic PS et al. (2006) Proc Natl Acad Sci U S A. |
| miR-668 | hypoxic injury | Qingqing Wei, Zheng Dong et al. (2018) J. Clin. Invest. |
| miR-708 | adaptive UPR signalling | Behrman S. et al. (2011) J Cell Biol. |
| miR-7a | cardiac injury  ER stress | Danielle E Read et al. (2014) Cell Biosci. |
| miR-92 | hypoxic injury | Licheng Gong, Haiming Xu et al. (2018) Cell. Physiol. Biochem. |
| miR-9-3p | brain and heart injury | Ogata K et al. (2015) Toxicol Pathol. |
| miR-98 | hypoxic injury | Chang-Lin Zhai, Song Zhang et al. (2019) IUBMB Life |

**FIGURE 3|** Circulating miRNAs significantly regulated in trauma hemorrhagic shock compared to sham group (p<0.01). Sham group (n = 4 animals); THS group (n = 8 animals).

**FIGURE 4|** Spearman´s correlations coefficients (r) between PCB and circulating miRNAs induced by THS. Sham group (n = 4 animals); THS group (n = 8 animals).

**FIGURE 5|** Circulating miRNAs significantly regulated by tunicamycin compared to sham group (p<0.01). Sham group (n = 4 animals); Tunicamycin group (n = 3 animals).
